# Structures of the Signal Recognition Particle Receptor from the Archaeon *Pyrococcus furiosus*: Implications for the Targeting Step at the Membrane

**DOI:** 10.1371/journal.pone.0003619

**Published:** 2008-11-03

**Authors:** Pascal F. Egea, Hiro Tsuruta, Gladys P. de Leon, Johanna Napetschnig, Peter Walter, Robert M. Stroud

**Affiliations:** 1 Department of Biochemistry and Biophysics, University of California San Francisco, San Francisco, California, United States of America; 2 Stanford Synchrotron Radiation Laboratory, Stanford Linear Accelerator Center, Stanford University, Stanford, California, United States of America; 3 Laboratory of Cell Biology and Howard Hughes Medical Institute, The Rockefeller University, New York, New York, United States of America; Massachusetts Institute of Technology, United States of America

## Abstract

In all organisms, a ribonucleoprotein called the signal recognition particle (SRP) and its receptor (SR) target nascent proteins from the ribosome to the translocon for secretion or membrane insertion. We present the first X-ray structures of an archeal FtsY, the receptor from the hyper-thermophile *Pyrococcus furiosus (Pfu)*, in its free and GDP•magnesium-bound forms. The highly charged N-terminal domain of *Pfu*-FtsY is distinguished by a long N-terminal helix. The basic charges on the surface of this helix are likely to regulate interactions at the membrane. A peripheral GDP bound near a regulatory motif could indicate a site of interaction between the receptor and ribosomal or SRP RNAs. Small angle X-ray scattering and analytical ultracentrifugation indicate that the crystal structure of *Pfu*-FtsY correlates well with the average conformation in solution. Based on previous structures of two sub-complexes, we propose a model of the core of archeal and eukaryotic SRP•SR targeting complexes.

## Introduction

Targeting and translocation of proteins across and into membranes is essential to all life forms. The process is mediated by evolutionarily related signal recognition particles (SRPs) and their cognate membrane-associated receptors (SRs also called FtsYs in Bacteria and Archaea) [1]. The core proteins of SRPs and SRs are GTPases that each contain a structurally and functionally conserved NG domain where the G domain adopts a *ras*-like fold responsible for GTP binding and the N-domain adopts a four α-helix bundle fold. In Archaea, the SRP is composed of two proteins, SRP54 (also called Ffh in Bacteria) and SRP19, and an SRP RNA. In addition to the NG domain, SRP54 contains a C-terminal methionine rich (M) domain that binds SRP RNA and provides the signal-sequence binding site; a flexible linker tethers this M domain to the NG catalytic core. SRP19 plays an architectural role in the stabilization of the SRP RNA and its interaction with SRP54. The SRP RNA is essential for survival [2], [3] and facilitates interaction between SRP and SR [4], [5].

SRPs sample polypeptide chains emerging from the ribosome and bind to those bearing a signal sequence that specifies secretion or membrane insertion. Targeting of the ribosome-nascent chain-SRP complex to the membrane embedded translocon is mediated through a dynamic GTP-dependent interaction between the NG domains of the SRP54 and the SR subunits. Structural studies have shown that the two GTPases interact tightly through the so-called “twinning” of their GTP substrates [6], [7]. At the membrane, upon reciprocal GTP hydrolysis the SRP•SR complex dissociates triggering transfer of the ribosome-nascent chain to the translocon. Although the mechanisms driving complex assembly have been elucidated, very little is known about an essential step of the targeting cycle: the transfer step. There is growing evidence of direct interaction between the SR and the translocon at the membrane in both bacterial and eukaryotic systems [8]–[10].

In contrast to their eukaryotic homologues, which are heterodimers containing a separate membrane anchoring subunit [11], [12], bacterial and archaeal receptors are composed of just the SR core protein, FtsY. While FtsY and some other bacterial receptors possess an extra N-terminal A domain of variable size and sequence, most bacterial and archeal receptors are further streamlined and reduced to the strictly conserved NG core. These “short” receptors, nevertheless, efficiently target the ribosome-nascent chain-SRP complex to the translocon, raising the question of what are the structural determinants for the membrane interaction.

Here we describe the X-ray structures and solution conformations of FtsY, the SR from the hyper-thermophilic archaeon *Pyrococcus furiosus* (*Pfu*), in its free and GDP•magnesium-bound forms. The unique features revealed by these structures, along with our recently reported structures of *Pfu*- SRP54 and SRP19 (*in press in PloS One*) have been incorporated into a model of the archeal SRP•SR targeting complex.

## Results

**Figure 1:**
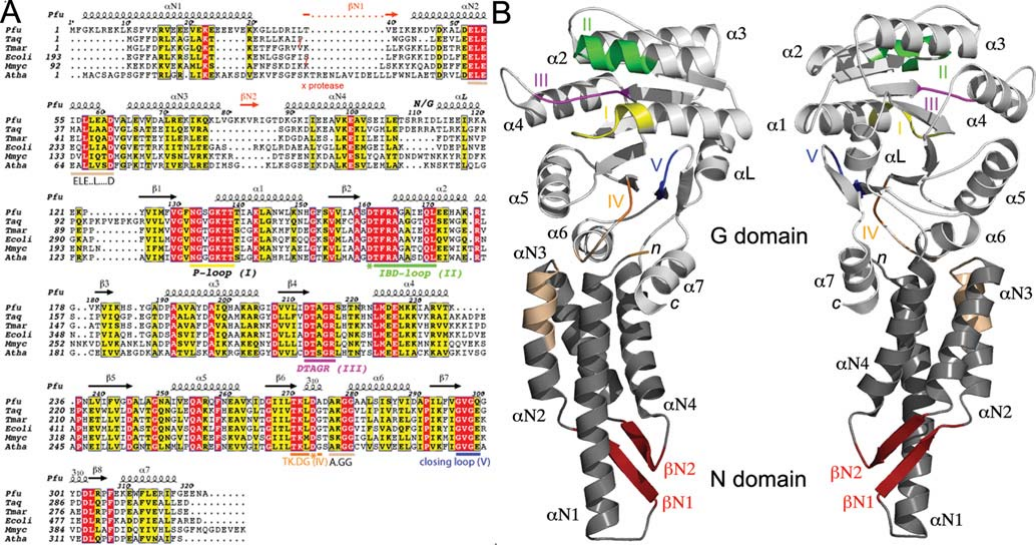
The FtsY from *Pyrococcus furiosus*. (A) Sequence alignment of FtsY/SRs of known structure including *Pfu*, *Thermus aquaticus (Taq)*, *Thermotoga maritima (Tma)*, *Mycoplasma mycoides (Mmyc)* and *Arabidopsis thaliana (Atha)* (chloroplast). The protease sensitive sites observed in *E.coli* and *Taq* FtsYs upon FtsY•Ffh complex formation are indicated (x). The ELEX_2_LX_3_D in the N domain is indicated. (B) Overall structure of the *apo* monomer of *Pfu*-FtsY. Two views related by a 180° rotation along a vertical axis are shown. The secondary structure elements of *Pfu*-FtsY are indicated. α-helices and conserved motifs of the SRP/SR-GTPases subfamily are labeled.

We crystallized and solved the X-ray structures of the *apo* and GDP•magnesium forms of *Pfu*-FtsY. The structures were solved at 2.2 and 2.0Å resolution for the *apo* and nucleotide-bound proteins, respectively (**Table 1** **and** **Material and Methods**). The *apo* receptor was crystallized in two different crystallization conditions in absence of guanine nucleotide and its structure solved *de novo* using single wavelength anomalous dispersion of selenium; the structure is therefore not biased towards any of the previously solved homologues. The overall structure of the *apo* receptor is shown in **Figure 1** with all sequence motifs characteristic of SRP GTPases well defined.

**Table 1:** X-ray data collection and structure refinement statistics.

Structure	*apo Pfu*-FtsY	*apo Pfu*-FtsY	*Pfu*-FtsY with GDP
PDB ID	3DM9	3DMD	3E70
**data set**	**ALS050904**	**ALS310806**	**ALS110508**
**data statistics**			
wavelength	0.97949Å	1.11588Å	1.11588Å
phasing method	Se-SAD	MR	MR
space group	P622	C2	C2
and	a = 144.6Å	a = 150.0Å	a = 113.1Å
cell dimensions	c = 70.5Å	b = 102.4Å	b = 53.0Å
		c = 101.8Å	c = 61.3Å
		β = 119.8°	β = 107.4°
AU content	1 molecule	3 molecules	1 molecule
solvent content	54%	63%	50%
resolution limits (last shell)	50.0−2.2Å (2.3−2.2Å)	88.4−2.2Å (2.3−2.2Å)	58.3−2.3 (2.1−2.0 Å)
unique reflections	21,908 (1,738)	65,992 (9,460)	23,541 (3,294)
redundancy	13.7 (11.3)	3.5 (3.1)	2.4 (2.3)
completeness	98.7% (96.1%)	98.1% (97.1%)	95.9% (92.6%)
*I/σ(I)*	14.9 (3.9)	8.9 (1.4)	8.7 (1.7)
*R_sym_*	9.3% (63.0%)	7.4% (76.6%)	4.9% (44.7%)
**refinement statistics**			
resolution range	35.9−2.2Å	65.1−2.2Å	29.7−2.0Å
reflections used work (test)	19,777 (2,000)	63,548 (2,000)	21,449 (1,999)
*R_free_/R_fac_*	23.9%/20.3%	24.6%/20.5%	26.3%/22.8%
overall figure of merit	0.906	0.897	0.825
overall B_wilson_	39 Å^2^	43 Å^2^	41 Å^2^
protein atoms	2,344 atoms, 45 Å^2^	7,417 atoms, 49 Å^2^	2,349 atoms, 62 Å^2^
ligand atoms	No ligand	No ligand	2 GDPs, 45 Å^2^ (100%) and 65 Å^2^ (69%)
	1 MPD, 47 Å^2^	6 glycerols, 61 Å^2^	1 magnesium, 45 Å^2^
	4 phosphates, 84 Å^2^	10 sulfates, 66 Å^2^	
solvent atoms	137 waters, 52 Å^2^	469 waters, 50 Å^2^	83 waters, 60 Å^2^
r.m.s.d. bonds	0.007 Å	0.006Å	0.016Å
r.m.s.d. angle	1.062°	0.939°	1.368°
Ramachandran Analysis			
residues in preferred regions	95.6%	97.3%	95.7%
residues in allowed regions	3.7%	2.4%	2.5%
outliers	0.7%	0.3%	1.8%

MR indicates phasing by molecular replacement. Se-SAD indicates phasing performed using single wavelength anomalous dispersion of selenium. AU stands for asymmetric unit.

r.m.s.d is the root-mean square deviation from ideal geometry.

*R_sym_* = Σ_hkl_Σ_i_ |*I*_hkl,i_−<*I*_hkl,i_>|/Σ_hkl_Σ_i_ |*I*_hkl,i_| where <*I*_hkl,i_> is the average intensity of the multiple hkl, i observations for symmetry-related reflections.

*R_cryst_* = Σ|*F*_obs_−*F*_calc_|/Σ|*F*_obs_|. *F*_obs_ and *F*_calc_ are observed and calculated structure factors, *R_free_* is calculated from a set of randomly chosen 5 to 10% of reflections, and *R_cryst_* is calculated over the remaining 90 to 95% of reflections.

The refined occupancies of 100% (catalytic GDP) and 69% (external GDP) and atomic displacement factors of the two GDPs are indicated.

### Two molecules of GDP are bound to the receptor

**Figure 2:**
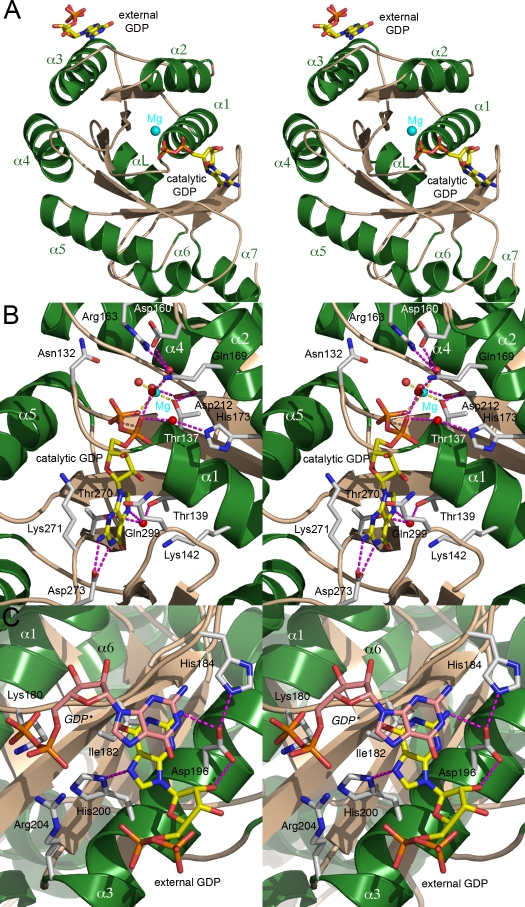
The two bound GDP nucleotides in the G domain. (A) General view showing the relative positions of the two GDP molecules. The two GDPs are about 31Å apart. (B) The GDP•magnesium bound in the active site is represented with its hydrated magnesium ion. (C) The external GDP (yellow) is bound near the insertion box domain. Another stacked external GDP (GDP*) molecule belonging to a symmetry-related molecule is also represented (light pink) to emphasize the crystal packing contact. In (B) and (C) the residues involved in the interaction with a nucleotide are labeled. Hydrogen bonds are shown.

We tried to co-crystallize *Pfu*-FtsY in presence of GTP. Although SRP-GTPases, especially the SR subgroup, are distinguished by their low intrinsic GTPase activity and nucleotide specificity [13], the crystal structure we obtained showed the presence of GDP•magnesium bound in the catalytic site suggesting that nucleotide hydrolysis took place during the course of crystallization. Identical crystals could be obtained in presence of GDP but not in presence of non-hydrolyzable GTP analogs. The resulting structure was solved at 2.0Å resolution by molecular replacement using the *apo* structure as template. Two bound GDP molecules were identified (**Figure 2A**) and placed in the initial experimental electron density maps. Refinement to consistent atomic displacement factors shows that the GDP observed in the cognate binding site is present at full occupancy while the external GDP is present at only 69% occupancy despite the fairly high concentration (10 mM) of nucleotide used for crystallization; this lower occupancy probably reflects the lower affinity of this binding site.

### The cognate nucleotide-binding site

In both *apo* structures, the guanine nucleotide-binding cleft contains either a phosphate or a sulfate ion that occupy the β-phosphate binding site observed in GDP- or GTP- bound forms [14] (**Supplementary** **Figure S1**). This position constitutes a substrate-anchoring site. The GDP in the catalytic site is accompanied by a hydrated magnesium ion (**Figure 2B**). The sidechains of two conserved aspartates, the catalytic aspartate Asp160, and Asp212, converge towards the β-phosphate of the GDP and the magnesium. The side-chain from the conserved Arg163 (motif II) contributes to electrostatic balance within the binding site. Upon GDP binding, motif IV shifts about 1.8Å bringing the carboxylic group of the conserved, nucleotide specifying Asp273 into position to establish two hydrogen bonds, 2.7Å and 2.9Å long, with the guanine ring nitrogen atoms N1 and N2. The guanine ring is also sandwiched between the sidechains of residues Lys271 (motif IV) and Gln299 from the closing loop that also undergoes a 1.8A shift. In presence of GDP, the conformation of the catalytic site represents an intermediate between the *apo* and ‘Ffh-bound’ FtsY conformation; a similar observation can be made when comparing the *Pfu* and *Taq* GDP-bound structures [15] (**Supplementary** **Figure S1**). In particular both structures show that the conserved DTAGR motif III is not locked in the conformation observed in the complex.

### A GDP bound at the surface next to the Insertion Box Domain (IBD)

The external nucleotide lays on a relatively flat surface delineated by residues Lys180, Ile182, His184, Asp196, His200 and Arg204 (**Figure 2C**). The nucleotide is bound at a crystal lattice contact with its purine ring stacked against its symmetry related molecule, the distance between the planes of the two stacked purine rings is about 3.2Å similar to the distance observed in a RNA helical chain. His200 is hydrogen-bonded with the N7 nitrogen from the guanine ring. The sidechains of Arg204 and Lys180 point towards the α and β phosphates of the stacked and symmetry-related nucleotide. Asp196 which hydrogen-bonds with the 2′OH of the ribose and the N2 guanine ring, is held in place by His184. This binding surface exhibits some degree of conservation. Asp196 is conserved in all receptors with the interesting exception of receptors belonging to the subgroup of chloroplast SRPs that do not involve an SRP RNA to mediate protein targeting [16]. This area maps next to the conserved IBD (motif II) specific to all SRP-GTPases.

### Clusters of charged aminoacids stabilize the N domain

**Figure 3:**
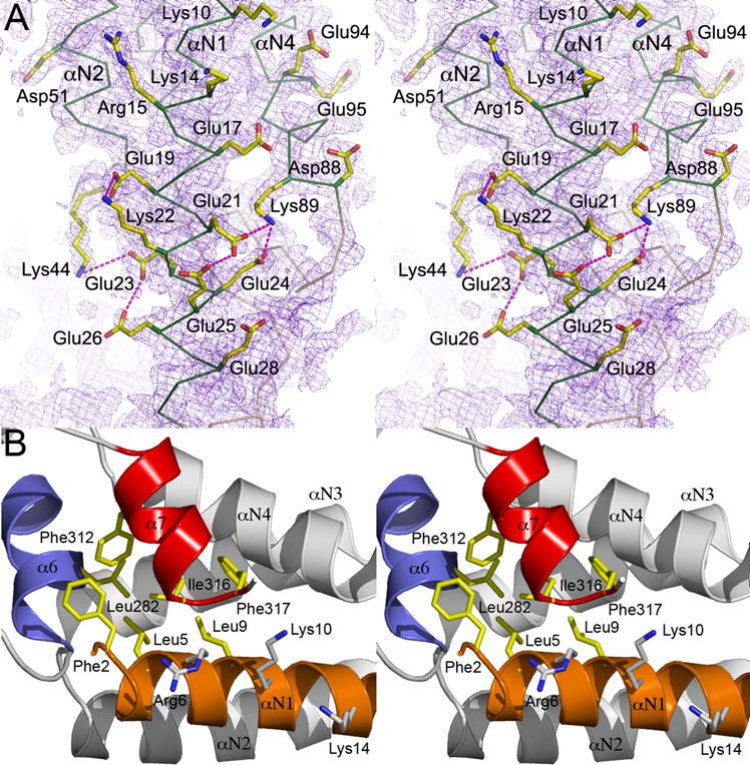
The N domain. (A) Stabilization of the highly charged N domain of *apo Pfu*-FtsY through an intricate network of salt bridges and hydrogen bonds. 2.2Å resolution likelihood-weighted *2mFo-DFc* Fourier difference electron density map contoured at 1.4σ showing the most solvent-exposed tip of the N domain. A subset of basic and acidic side-chains is shown. (B) Properties of the N-terminal helix αN1 of the N domain. The helix αN1 packs tightly against the G domain helices α6 and α7 (C-terminal helix). Conserved hydrophobic residues contributing to the interaction between the N-terminus of the N domain and the C-terminus of the G domain at the N/G interface are labeled. Solvent exposed basic residues are also indicated. Helices are labeled. These properties appear to be conserved in all archeal receptor sequences.

The N domain of *Pfu*-FtsY is very rich in charged residues (30 acidic and 25 basic residues out of a total of 110 residues representing 50%). These residues contribute to the high thermo-stability of *Pfu*-FtsY through an intricate network of intra-molecular salt bridges and hydrogen bonds that stabilize the overall fold of the N domain (**Figure 3A**). At the C terminal end of helix αN1 the carboxylate groups from residues Glu21 and Glu24 interact with the amino group of Lys89 of helix αN3. In a similar fashion, Glu23 on helix αN1 interacts with Lys44 from helix αN2. Such extended ion-pair networks contribute to thermostability in proteins [17]. The N-terminus of helix αN1 is characterized by solvent exposed basic residues (**Figure 3B**) and packs tightly against the G domain; in particular with helices α6 and the C-terminal helix α7. Sequence analysis suggests that these features are conserved throughout all archeal receptors (**Supplementary** **Figure S2**). These clusters of solvent-exposed basic residues on one face of αN1 and the surface of the N domain seem to be in an ideal position for either membrane anchoring, for example, through lysine or arginine “snorkeling” to negatively charged phospholipidic head groups, or for interaction with the ribosomal and/or the SRP RNAs.

### Compared to other SRs, the *Pfu* N-domain is unusual and highly elongated

**Figure 4:**
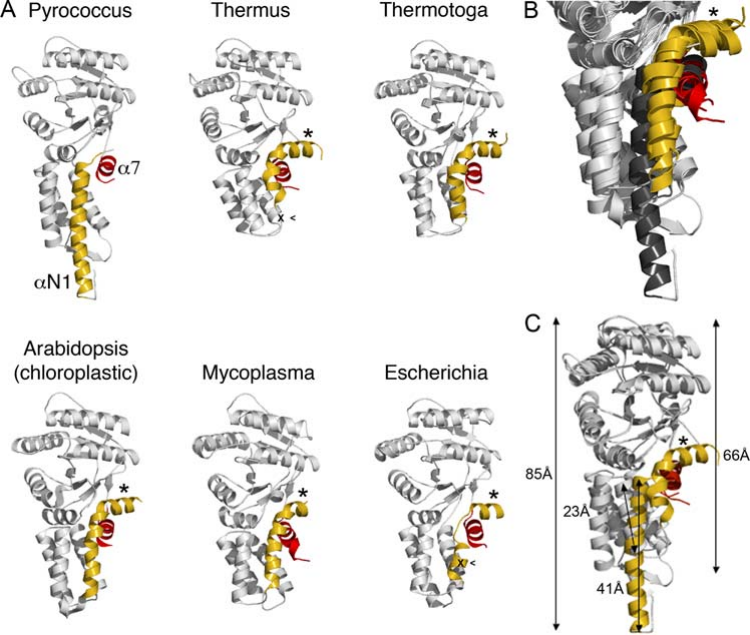
Comparison of the different SRP receptors. (A) The six available FtsY structures shown in the same relative orientation. In each case the helix αN1 in the N domain and the α7 C-terminal helix in the G domain are colored in yellow and red, respectively. An asterisk indicates the N-terminal extension observed in all FtsYs except *Pfu*. The sites of proteolysis observed in *E.coli* and *Taq* FtsYs upon Ffh•FtsY complex formation are also indicated (x). (B) Close-up view of the overlaid six FtsY structures showing the αN1 and α7 terminal helices. (C) Superposition of the *Pfu* and *Taq* FtsYs emphasizing the shape anisotropy of *Pfu*-FtsY due to the long αN1 terminal helix in its N domain. Distances along the longest axis of inertia are indicated.

To date structures of six SRs have been reported; these include the bacterial receptors, [18], [19], *Taq* [20], *Tma* [21], *Mmyc* [14], and the eukaryotic organelle-specific chloroplastic SR from *Atha* [22], [23]. Our *Pfu*-FtsY structure is the first representative from the Archaea kingdom. The *Pfu*-FtsY N domain is unusual in several aspects. While it retains the canonical four α-helical bundle fold observed in all SRP GTPases, it has an additional two-stranded anti-parallel β-sheet not seen in the other FtsY structures; βN1 is inserted between helices αN1-αN2, and βN2 is inserted between αN3-αN4 (**Figures 1A**). βN1 and βN2 assemble together to form a flat surface exposed at the tip of the N domain (**Figure 1B**). The αN1 helix of *Pfu*-FtsY, whose N terminus is perfectly defined, is 44Å-long and is a single secondary structure element with no bending or disorder. This helix protrudes out of the N domain (**Figure 1B** **and** **4A**). While the position of the C-terminal helix α7 is conserved in all SRs (Figure 4B), helix αN1 of the non-archeal homologues is bent, resulting in an N terminal extension that packs against the surface of the N/G domain including C terminal helix α7 (**Figures 4A and 4B**). The αN1 helix of *Pfu*-FtsY is not bent and its axis is shifted towards the core of the four α-helix bundle resulting in an overall more compact, albeit extended, N domain.

### The average solution conformation observed by SAXS agrees with the crystal structure

Compared to *Pfu*, *Taq*-FtsY is characterized by a short and compact N domain (**Figure 4C**). Both receptors can be modeled as prolate ellipsoids, similar in their short semi-axes but markedly different in their long semi-axes. Based on the X-ray structures the longest dimensions in the *Pfu* and *Taq* receptors are 91Å and 73Å, respectively. This difference is entirely due to the shape of the N domain. SAXS (Small Angle X-ray scattering) and AUC (Analytical Ultracentrifugation) (**Material and Methods**) allowed us to compare the relative shape anisotropy of the two receptors in solution and validate the differences observed between the two receptors as revealed by the X-ray structures.

**Figure 5:**
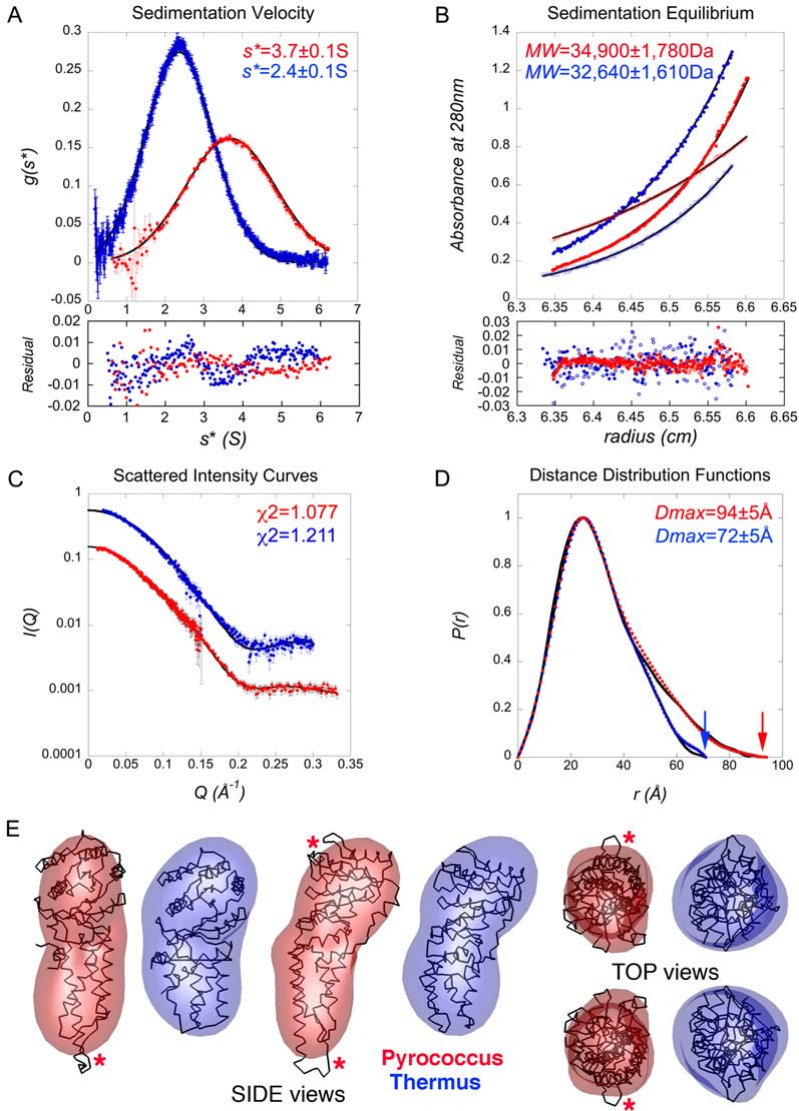
AUC and SAXS characterization of the *Pfu* and *Taq* FtsYs. *Pfu* data are represented in red and *Taq* in blue. (A) Sedimentation velocity analysis. Data are presented in g(S*) format using the time-derivative method analysis. The curves fitted against Gaussian functions (solid lines) are shown with their corresponding residual deviations. (B) Sedimentation equilibrium analysis. Radial distributions of concentrations along with the fitted exponential curves (solid lines) are plotted with their corresponding residual deviations. Two representative fits are shown for each protein. In all cases curves were fitted assuming a single species system. (C) and (D) High-angle analysis of SAXS data. (C) Scattered intensity curve fits. The experimental *I(Q)* curves (circles) displayed with errors bars were fitted using *CRYSOL* against the theoretical curves (solid lines) calculated from the crystal structures. (D) Distance distribution analysis. The experimental distance distribution functions *P(r)* are displayed (circles) with errors bars and compared with the ones calculated (solid lines) from the crystal structures using *CRYSOL* and *GNOM*. (E) *Ab Initio* 3D shape restoration by simulated annealing minimization. The protein envelopes are described using spherical harmonics expansion (fifth order). The retrieved shapes are rendered with *ASSA* [52] (drawn as transparent envelopes) with their corresponding crystal structures after optimal superposition along their respective inertia axes using *SUPCOMB* [53]. The final χ^2^s are 0.943 and 1.109 for *Pfu* and *Taq* receptors respectively. Four different orientations are shown to emphasize the quality of the reconstructions. Red asterisks indicate the areas of poorest overlap that correspond to the disordered regions in the crystal structures.

The apparent sedimentation coefficients of *Pfu*-FtsY (*s* = 3.7±0.1S) and *Taq*-FtsY (*s* = 2.4±0.1S) were determined (**Table 2** **and** **Figure 5A**) and both receptors appeared as monomers in solution. The apparent monomeric association state established by velocity sedimentation was rigorously confirmed by equilibrium sedimentation experiments carried out over a wide, but still dilute, range of protein concentrations. Equilibrium experiments yielded molecular weight estimations of 34,900±1,780 Da and 32,640±1,610 Da for *Pfu* and *Taq*, respectively, in good agreement with the calculated values of 35,810 Da and 33,055 Da (**Table 2** **and** **Figure 5B**).

**Table 2:** Solution study by Analytical Ultracentrifugation and Small-Angle X-ray Scattering.

Method	Analytical Ultracentrifugation Sedimentation Velocity	Analytical Ultracentrifugation Sedimentation Equilibrium	
		experimental *Mr*	calculated MW
*apo Pfu*-FtsY	*s* = 3.7±0.1S	*Mr* = 34,900±1,780Da	MW = 35,810Da
*apo Taq*-FtsY	*s* = 2.4±0.1S	*Mr* = 32,640±1,610Da	MW = 33,055Da

Method	SAXS (Guinier analysis)		SAXS (Distance distribution analysis)	
	experimental *R_G_*	calculated *R_G_*	experimental *R_G_* and *D_max_*	calculated *D_max_*
*apo Pfu*-FtsY	*R_G_* = 25.8±0.2Å	*R_G_* = 25.4Å	*R_G_* = 25.6±0.2Å *D_max_* = 94±5Å	*D_max_* = 91±5Å
*apo Taq*-FtsY	*R_G_* = 22.5±0.2Å	*R_G_* = 22.6Å	*R_G_* = 22.8±0.2Å *D_max_* = 72±5Å	*D_max_* = 71±5Å

SAXS was used to investigate the oligomeric state, size and molecular shapes of the two receptors. A concentration dependence study of the apparent radius of gyration was done from concentrations ranging from 2.5 to 25 mg. ml^−1^ in the small angle region (Guinier analysis). All solutions appeared to be monodisperse with Guinier plots linear over an appropriate angular range (0.5<*QR_G_*<1.25) (data not shown). There were no signs of protein aggregation or association over the concentration range used in this study. Solutions of *Pfu*- and *Taq*- FtsYs thus behaved ideally with little effect of protein concentration on the apparent radius of gyration as measured by SAXS.

The experimental intensity curves were fitted against the theoretical curves calculated from the X-ray structures using *CRYSOL* [24] with adjustment of the contribution due to the hydration shell (**Material and Methods**). The resulting fits (**Figure 5C**) are of good quality with respective *χ^2^* values of 1.077 and 1.211 for *Pfu*- and *Taq*- FtsYs. The corresponding pair-distance distribution functions *P(r)* derived from experimental or theoretical intensities were determined by Fourier transformation using *GNOM* [25] and superimposed (**Figure 5D**). Based on the experimental *P(r)*, the maximum distance values of *D_max_* = 94±5Å and *D_max_* = 72±5Å, for *Pfu* and *Taq*, respectively, are in very good agreement with those of 91Å and 71Å derived from the corresponding X-ray structures (**Table 2**). Calculation of the distance distributions also allowed estimation of the radius of gyration independently from the Guinier analysis (**Material and Methods**). The experimental values for the radius of gyration of *R_G_* = 25.6±0.1Å and *R_G_* = 22.8±0.1Å, for *Pfu* and *Taq* respectively, are in very good agreement with those of 25.4Å and 22.6Å calculated from the X-ray structures (**Table 2**). The experimental values of molecular dimensions obtained from the analyses in the low (*R_G_*) or high angle (*R_G_* and *D_max_*) regions are in close agreement with those obtained using the X-ray structures. Thus our solution data show that the average conformations adopted by the two receptors in solution are similar to those observed in their crystalline environments and that the relative shape anisotropy of *Pfu*-FtsY is due to its long and extended N domain.

The low-resolution structures of *Pfu*- and *Taq*- FtsYs were restored using the *ab initio* simulated annealing procedure implemented in *DAMMIN* [26] (**Material and Methods**). This approach was used to independently assess the average conformation adopted in solution by the two receptors. The reconstructed shapes are very close to the crystallographic envelopes (**Figure 5E**). Superposition of the reconstructed shapes with crystal structures show that the main differences lay in the region corresponding to the N domain. The shapes of both receptors were faithfully restored and agree well with their respective crystallographic *apo* structure. Superposition of the SAXS-derived envelope and the crystal structure also suggests that the apical part of the N domain is more dynamic, as indicated by the slight lack of overlap between the SAXS reconstruction and the most solvent-exposed extremity of the αN1 helix. These results correlate well with the different crystal structures that show that the loops connecting helices αN1-αN2 and helices αN3-αN4 are more dynamic and in some cases disordered.

### Relative conformations of the N and G domains and formation of the FtsY•SRP54 complex: Implications for the SRP-dependent protein-targeting cycle

**Figure 6:**
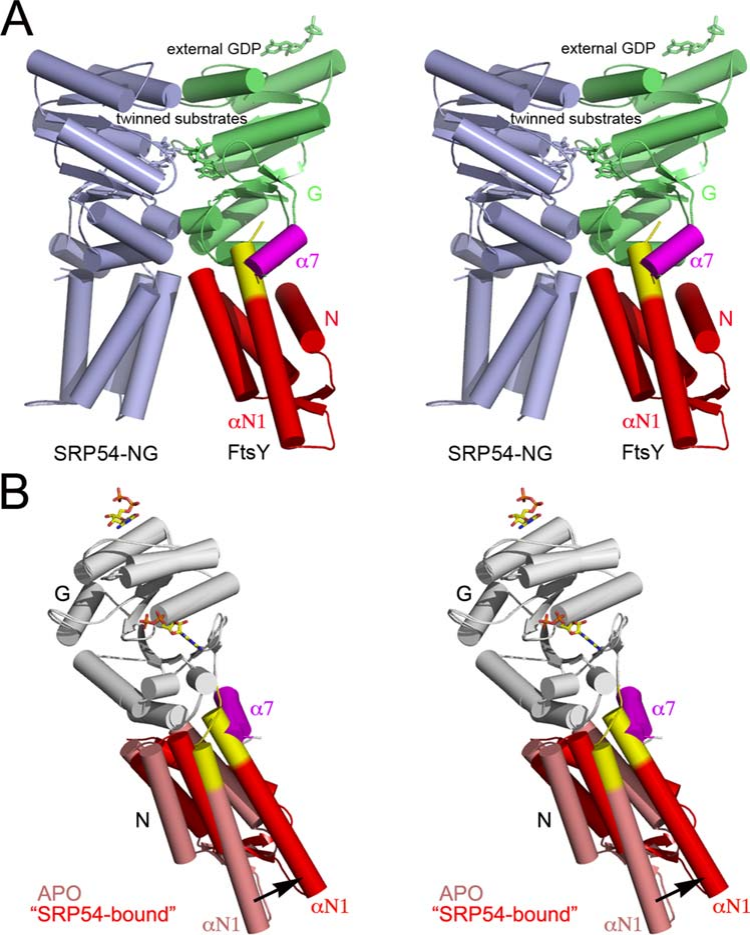
Model of domain rearrangement in *Pfu*-FtsY upon its interaction with SRP54. (A) The overall structure of the *Pfu* model is based on the structure of the *Taq*-FtsY•FfhNG. For *Pfu*-FtsY, the N and G domain have been colored red and green respectively except for the αN1 and α7 terminal helices highlighted in red and pink. The stretch of basic aminoacids present at the N terminus of αN1 (yellow) clashes with α7. For the N domain, the DX_4_ELEX_2_LX_3_D motifs (Glu47-Asp61 (*Pfu*) on Glu29-Asp43 (*Taq*)) were aligned. The entire G domains (Val125-Phe307 (*Pfu*) on Val104-F292 (*Taq*)) with the omission of the C-terminal α7 helix were aligned. A similar alignment was made for the *Pfu*-SRP54 NG (light blue) including Leu39-Asn45 (*Pfu*) on Leu38-Asn44 (*Taq*) for the N domain, and Val106-Phe284 (*Pfu*) on Val104-Phe282 (*Taq*) for the G domain. The *Pfu*-SRP54 structure (pdb code 3DM5) used for modeling is reported in a previous article (*in press in PloS One*). The N-G linkers were omitted. (B) Detail showing the rearrangement undergone by the αN1 and α7 terminal helices at the N/G interface upon complexation.

In the FtsY•SRP54 complex the interface involves both N and G domains: In particular, the N domain ELEX_2_LX_3_D motifs present in both SRP54 and FtsY (see the sequence alignment in **Figure 1A**) come in close contact upon complex formation. A model of the *Pfu*-FtsY•SRP54 complex was assembled based on our structure of the *Taq* complex. The N and G domain were aligned independently to generate an NG conformation similar to the one observed in the *Taq* complex (**Figure 6A**). In the case of *Pfu*-FtsY, the N domain has to undergo a rotation and translation to adopt the complexed conformation (**Figure 6B**). This rotation causes the terminal helices αN1 and α7 to clash, highlighting the requirement for a substantial displacement of αN1. The extent of such steric hindrance is likely to be more important than our model suggests, since the *Taq* complex structures have shown that α7 repacks more tightly against the NG core interface upon complex formation. The area of overlap maps to the stretch of solvent-exposed basic aminoacids in αN. A recent structure of GDP-bound *Taq*-FtsY with its αN1 helix deleted revealed that this truncated version of the receptor adopts a conformation close to the one observed in the *Taq* complex [15].

### A model for the interaction between SRP and SR in the archeal and eukaryotic core of the targeting complex

**Figure 7:**
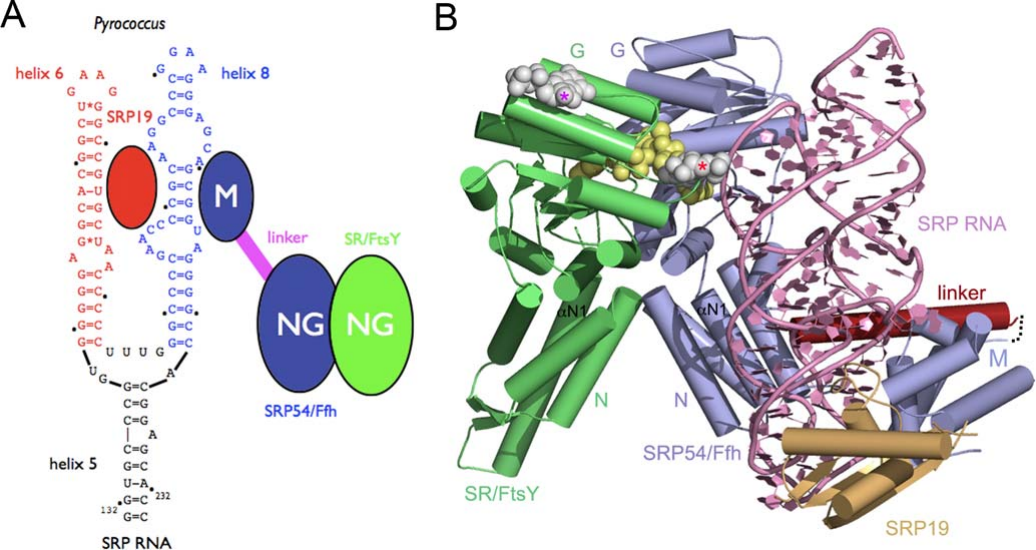
A model for the association between the SRP and it receptor SR in the targeting complex. (A) Schematic showing the overall organization of the *Pfu* targeting complex. The core of the SRP RNA is shown with helices 6 and 8, the respective binding sites for the proteins SRP19 and SRP54. In SRP54 the M domain, responsible for both SRP RNA and signal sequence recognition, is connected to the NG domain (GTPase) through a flexible linker (red). Although the NG of SRP54 domain has also been shown to interact loosely with the core of the SRP RNA, for the sake of clarity this is not represented on this schematic. (B) The *Pfu* protein structures were used to generate this model based on the *Taq*-FtsY•FfhNG and *Mja*-SRP complexes structures. FtsY, SRP54 and SRP19 are colored in green, blue and orange respectively. The core of the *Mja*-SRP RNA, composed of helices 5,6 and 8 is represented in pink. In the SRP54 subunit, the GM linker, colored in red, has been manually repositioned. Nucleotides are represented using space-filling models. At the FtsY•SRP54 interface, the twinned GTP substrates are colored in yellow and the two external nucleosides observed in the *Taq*-FtsY•FfhNG structure bound to GDP-AlF_4_ (red asterisk) and in the *Pfu*-FtsY structure (magenta asterisk) are colored in white. αN1 helices are labeled. The *Pfu*-SRP19 structure (pdb codes 3DLU and 3DLV) used for modeling has been reported in a previous article (*in press in PloS One*).

The targeting complex is formed when SRP interacts with its receptor (**Figure 7A**). A functional archeal SRP is organized around two proteins, SRP19 and SRP54 that assemble on SRP RNA. We have also recently reported the structures of the SRP54 and SRP19 from *Pfu* (*in press in PloS One*). The present structure of the associated receptor complements this work. *Pfu* is the first organism where separate structures of all of the proteins present in the targeting complex are available at high resolution. We generated a model of this complex, based on three FtsY•FfhNG heterodimer structures from *Taq* [6], [7], [27], [28] and the SRP structure from *Methanococcus jannaschii (Mja)* [29]. In the model, we superposed the *Pfu*-FtsY (with its two GDPs) and the *Pfu*-SRP54 NG domain onto the *Taq*-FtsY•FfhNG structure to generate the equivalent *Pfu*-FtsY•FfhNG interface (**Figure 6A**). The NG domain of *Mja*-SRP was superposed on the FtsY•FfhNG core to model the relative position of the SRP RNA. The *Pfu*-SRP19 subunit and the *Pfu*-SRP54 M domain (with the omission of the G-M linker) were then docked, assuming similar, but not necessarily identical relative configurations of the NG and M domains in the SRP and the SRP•SR complexes (**Figure 7B** **and Supplementary** **Movie S1**).

## Discussion

We describe the X-ray structure of FtsY, the SR from the hyper-thermophilic archaeon *Pfu*, in its free and GDP•magnesium bound states. The *Pfu*-FtsY construct used in this study encodes the full-length receptor. The low resolution, solution scattering data complements our high-resolution crystallographic analysis and shows that the crystallographic structure and the average conformation adopted in solution are similar. While the overall architecture of the archeal receptor resembles its previously described bacterial homologues from the SRP/SR GTPases family, the structure of *Pfu*-FtsY reveals novel features: The elongated N domain lacks the N-terminal extension observed in all other bacterial FtsYs and is instead characterized by a long N-terminal helix αN1 that packs against the NG core in a different way; sequence analysis suggests that archeal receptors may cluster apart from their bacterial homologues.

This is the first structure of a full-length GDP•magnesium bound SR. The previously reported *Taq*-FtsY•GDP structure was obtained with a receptor that lacked the first 20 aminoacids [15] and contained no magnesium, which is required for the association between FtsY and Ffh. [14]. The structure reveals two nucleotide molecules, one in the catalytic site and one located at the surface of the protein next to the IBD motif unique to all SRP-GTPases. The GDP•magnesium bound receptor adopts a conformation close to the one observed in the *Taq*-FtsY•Ffh complex.

Several studies on *E.coli* FtsY have shown that the A domain is involved in membrane anchoring [30], interaction with the translocon, and possibly regulation of the GTPase cycle. In particular, a positively charged, cleavable N-terminal sequence is involved in membrane binding [31]. The A domain is important for the initial attachment to the membrane; however, once bound its proteolytic cleavage from the NG catalytic core does not affect receptor function [32], [33]. Subtle changes introduced at the boundary between the A and the N domain [19], [34], also seem to greatly affect the ability of the receptor to interact efficiently with the membrane and target ribosomes. In *E.coli* the N-terminal extension of the N domain also plays a role in membrane interaction [19]. In *Pfu*-FtsY, the N-terminus of helix αN1 is positively charged and could constitute the primary membrane interaction site supporting initial attachment of the targeting complex to the membrane and/or interaction with the membrane-embedded translocon. Similar properties of the N-terminal extensions present in the *Mmyc* and *Tma* FtsYs have been described [14].

The N and C terminal ends of all SRP/SR GTPases are closely apposed and are proposed to play a regulatory role in the SRP-dependent targeting cycle [15]. In the case of FtsY, the rotation of the G domain relative to its C-terminal α7 helix and its N domain is coupled with the displacement of the N-terminal extension. Upon complex formation systematic proteolysis of the N-terminal helix of the *Taq*-FtsY and unfolding of the N-terminus of its partner Ffh have been observed *in vitro* [35] and in all *Taq*-FtsY•FfhNG complex structures. In *E.coli* and *Taq* FtsYs the proteolysis sites have been mapped at position Ser216 and Pro23 respectively. It remains unclear whether this alteration of the N-terminus of the receptor is a required step during SRP-mediated protein targeting. A recent study of *E.coli* FtsY and Ffh has shown that truncation of the first helix of the N domain of both proteins dramatically accelerates complex formation; the αN1-truncated Ffh and FtsY interact at nearly the RNA-catalyzed rate in the absence of the SRP RNA [36]. Furthermore, in the case of FtsY in presence of GMPPNP, NMR experiments showed that truncation of helix αN1 mimics the conformational changes associated with FtsY•Ffh complex formation [36]. Such results have not been transposed yet to either archaeal or eukaryotic systems that exhibit an increased level of structural complexity. However, in the model of the *Pfu*-FtsY•SRP54 complex that we present here, truncation or some other displacement of the N-terminus of αN1 is required for the FtsY to achieve the NG conformation observed in the *Taq*-FtsY•Ffh complex; these findings seem transposable to the SRP54 partner although the presence of the linker connecting the G and M domains complicates analysis.

We have built a model of the core of the archeal targeting complex based on structures of the *Taq*-FtsY•Ffh complex and an archeal SRP. Our model places SRP RNA on the same side of the FtsY•SRP54 core as a structural model of the *E.coli* targeting complex inferred from chemical footprinting [37]. Both models introduce asymmetry in the otherwise symmetric heterodimeric catalytic core. An external nucleotide-binding site identified in the *Taq*-FtsY•FfhNG complex [27] is adjacent to one of the two exits of the catalytic chamber. Residues from both Ffh and FtsY contribute to this external site that exhibits conserved sequence and structural features. In our model, this site maps on the FtsY•SRP54 interface that faces the SRP RNA. Our FtsY structure identified a second and distinct external nucleotide-binding site located on the surface of the receptor. In our model, this site represents a potential site of interaction with ribosomal RNA or regions of the SRP RNA that are not present in the models or available structures. Altogether, this suggests that a potential direct interaction with the SRP RNA could regulate the activity of the receptor in the targeting complex. The αN1 helix of the receptor is freely accessible to promote interactions with the membrane and/or the translocon, while the equivalent region in SRP54 is not accessible because of its vicinity with the SRP RNA. If the latter were also to regulate membrane interaction, the SRP RNA would have to move away.

The N-terminus of SRP receptors appears to play a crucial role in the assembly of the targeting complex and its regulation, while the symmetrical arrangement of the two SRP-GTPase twins seems to be mirrored in the conformational changes observed in their N termini. The SRP RNA has been shown to control a conformational switch regulating the interaction between the two SRP GTPases. As signal sequences bind to SRP54/Ffh in presence of SRP RNA, and the catalytic core of the targeting complex undergoes the structural changes priming it for interaction with the membrane and/or the translocon, the SRP RNA is likely to coordinate those events by regulating the activity of the receptor. This attractive hypothesis awaits further structural evidence.

## Materials and Methods

### Protein Expression and Purification

The gene encoding full-length *Pfu*-FtsY (PF1766) was amplified by PCR using genomic DNA and cloned in the pET28b vector (Novagen). The corresponding protein expressed as a fusion with a N-terminal hexahistidine tag cleavable with thrombin. Protein was expressed in BL21(DE3)-rosetta2 *E.coli* cells grown in auto-induction media [38] and seleno-substituted protein was expressed in B843(DE3)-rosetta2 *E.coli* cells grown in minimal media with glucose as carbon source and using the aminoacid pathway starvation method [39]. Purification was achieved in four steps combining heat selective precipitation, cobalt-chelating affinity chromatography, gel filtration and ion-exchange chromatography after removal of the purification tag. No detergent was used during purification or crystallization. The *Taq*-FtsY was expressed and purified as described previously [6], [20].

### Protein Crystallization

For crystallization, protein was concentrated at 20 mg. ml^−1^. Crystals of *apo Pfu*-FtsY were obtained at room temperature from a variety of conditions in hanging drops by the vapor diffusion method using a Mosquito nanoliter-scale robotic workstation (TTP Labtech). Two crystals forms were obtained for the *apo* protein. The hexagonal form (space group P622) grew in 1.1–1.5 M ammonium phosphate and 100 mM sodium acetate pH 5.0. The monoclinic form (space group C2) grew in 0.9–1.2 M lithium sulfate, 0.4–0.6 M ammonium sulfate and 100 mM sodium citrate pH 5.0. For the GDP-bound structure, soaking crystals of *apo Pfu*-FtsY failed but co-crystallization in presence of 10 mM GTP yielded several crystallization conditions. Best crystals grew in 14–17% PEG 8000 and 100 mM Tris pH 8.0 and belong to the monoclinic space group C2.

### X-ray Data Collection and Structure Determination

X-ray diffraction data were collected at beamline 8.3.1 at the Advanced Light Source (Berkeley, California) on Quantum 210 or 315r CCD detectors. The hexagonal crystals of *apo Pfu*-FtsY cryo-protected in 2-methyl-2,4-pentanediol diffracted to 2.2Å resolution. The monoclinic crystals of *apo Pfu*-FtsY cryo-protected in glycerol diffracted to 2.2Å resolution. The monoclinic crystals of *holo Pfu*-FtsY cryo-protected in ethylene glycol diffracted to 2.0Å resolution. Data were indexed, reduced and scaled with *HKL2000* [40] or *MOSFLM* [41] and *Scala* [42] using *Elves* [43]. The hexagonal form of *apo Pfu*-FtsY was solved using anomalous dispersion of selenium. SAD phasing and density modification were performed in *Phenix* [44]. Following location of the four expected seleniums, the figure of merit of 0.39 was further improved to 0.61 after density modification. The monoclinic form of *apo Pfu*-FtsY and the GDP•magnesium-bound structure were solved using the hexagonal structure as search model for molecular replacement in *Phaser* [45]. Partial automatic building and refinement were done using *Phenix* without use of non-crystallographic symmetry restraints in the case of the monoclinic crystal form. Model building was done in *Coot* [46]. In all structures the region encompassing residues T219-N221 following the conserved DTAGR motif (motif III) is poorly defined. The hexagonal form *apo* structure lacks residues 21–40 and 86–89. The hexagonal *apo* structure and the *holo* structure lack residues 25–37 and 85–89 at the tip of the N domain. For the GDP•magnesium bound structure, two GDP molecules were introduced and their relative occupancies refined to consistent atomic displacement parameters. TLS-refinement was also used for the *holo* structure by considering two separate groups encompassing the N domain (residues 1–110) and the G domain (residues 111–318). Structure qualities were assessed with *MolProbity* [47].

### Analytical Ultracentrifugation

#### Sample preparation and data measurement

For AUC experiments, the top-peak fractions from gel filtration were diluted as required and used immediately. Centrifugation was carried out in buffer, 20 mM Hepes pH 7.5, 250 mM KCl, 0.5 mM EDTA and 5 mM MgCl_2_, at 20°C using a Beckman Optima XL-A (Beckman Instruments Inc., Palo Alto, CA) with absorbance monitoring at 280 nm. Protein concentrations were in the range of 0.35–3.5 mg. ml^−1^, corresponding to molar concentrations of 10 to 100 µM. Partial specific volumes of proteins were calculated using amino-acid compositions; the values used for data analysis were 0.7307611 cm^3^. g^−1^ and 0.732851 cm^3^. g^−1^ for *Pfu*- and *Taq*- FtsYs, respectively.

#### Sedimentation velocity

The net sedimentation behavior of macromolecules is described by the Svedberg equation. For a species with a sedimentation coefficient *s*:
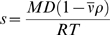
where *M* is the molecular weight, *D* the diffusion coefficient and *ν̅* the partial specific volume of the solute in a solvent of density *ρ*. The apparent sedimentation coefficients s* at the boundary can be determined using time-derivative analysis methods allowing derivation of the sedimentation coefficient distribution function g(s*) as implemented *DCDTplus* program [48]. To achieve the best hydrodynamic resolution, samples were spun at 50 000 rpm. Consecutive scans were recorded at regular intervals until complete depletion of the solute. Distribution functions were fitted against a Gaussian function.

#### Equilibrium sedimentation

At equilibrium, the concentration distribution generally approaches an exponential; for a mixture of non-interacting and ideally-sedimenting solutes, *a(r)* the measured absorbance as a function of the radial position *r* can be formalized as [49]:
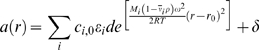
where the summation is over all *i* species; *c_i,0_* represents the molar concentration of species *i* at a reference position *r_0_*; *ω*, *M_i_*, 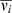 and *ε_i_* respectively represent the angular velocity, the molar mass, partial specific volume, and extinction coefficient; *d* is the optical path length and *d* a baseline correction accounting for differences in non-sedimenting solutes between sample and reference and small non-idealities in cell assembly and data acquisition. Samples (3 to 9 concentrations) were spun at 4 different speeds (8500, 12 000, 17 000 and 20 000 rpm). Data were analyzed in *WinNonLin* (from David Yphantis at http://spin6.mcb.uconn.edu/winnonlin/winnonln.html) using non-linear least squares analyses and assuming a single component model. Determination of the reduced molecular weights (*σ*) yielded the molecular weights.

### Small Angle X-ray Scattering

#### Sample preparation and data collection

For SAXS experiments, the experimental buffer was 20 mM Hepes pH 7.5, 250 mM KCl, 0.5 mM EDTA and 5 mM MgCl_2_ and 10 mM DTT. For measurements at low *Q*, the top-peak fractions from gel filtration were used without further concentration (concentration range 2.5–12.5 mg. ml^−1^). For measurements at high *Q*, samples were concentrated up to 25 mg. ml^−1^. SAXS data were recorded at beam line BL4-2 [50] at the Stanford Linear Accelerator (Stanford, USA). Samples contained in 1.2 mm path cells with thin mica windows were thermostated at 15°C. The X-rays wavelength was λ = 1.38Å. For *Taq*-FtsY, scattered X-rays were detected using one-dimensional position sensitive proportional counters. The short distance setup for the high-angle analysis with a sample-to-detector distance of 960 mm and a He_2_/CO_2_ gas-filled detector corresponded to an angular range of 0.018Å^−1^<*Q*<0.30Å^−1^. The long distance setup for the small-angle analysis with a sample-to-detector distance of 1960 mm and an Ar_2_/CH_4_ gas-filled chamber detector corresponded to an angular range of 0.009Å^−1^<*Q*<0.175Å^−1^. For *Pfu*-FtsY, scattered X-rays were recorded using a MarCCD165 detector using two different detector-to-sample distances (0.5 and 2 m). For each sample or buffer, 30 frames of 30 seconds were recorded, individually inspected to check for X-ray induced sample damage. The *Q*-axes of the detectors were calibrated using the {1,0,0} and related reflections of a cholesterol myristate powder sample.

#### Data analysis and processing

Individual scattering curves were normalized to the incident beam intensity, corrected for background and radially averaged using the programs *Otoko*, *Sapoko* and *MarParse* [50]. Two scattering curves, one recorded at low concentration and low angle and one recorded at high concentration and high angle region, were merged and scaled together using the program *GNOM* before calculation of the distance distribution function or fit against a theoretical scattering curve. No geometrical corrections were applied on experimental curves.

#### Guinier analysis

The data in the lowest angle range when plotted as ln*I(Q)* versus *Q^2^* give the radius of gyration *R_G_* and *I(0)* the forward scattering intensity extrapolated at zero angle with:
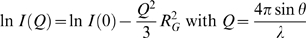

*Q* is the scattering vector for a scattering angle of *2θ*. For a sphere, this expression is valid in a *QR_G_* range up to 1.3 which can be extended up to 2 in some cases, the most conservative limit being in the range of *QR_G_* = 1. The values of *I(0)* allow the calculation of an apparent molecular mass *M_r_* of the particle in solution as far as the solute concentration has been determined accurately. Guinier analyses were performed using the program *PRIMUS* [51].

#### Distance distribution function P(r)

Indirect transformation of the scattering intensity *I(Q)* in reciprocal space into that in real space were carried out using the program *GNOM* [25] since:
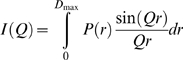
As *P(r)* corresponds to the distribution of distances *r* between any two volumes elements within one particle, it offers an alternative calculation of *I(0)* its zero^th^ moment, *R_G_* its second moment and gives also *D_max_* the chord or maximum dimension of the macromolecule:
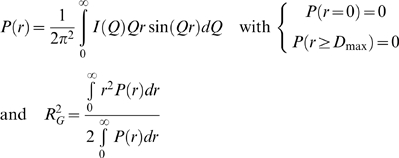
As calculation of *P(r)* includes an estimate of the chord, *D_max_* was determined from the values of *r* when *P(r*) became zero at larger *r* values; a range of maximum chords was systematically tested for integration and the final choice of *D_max_* was based on four essential criteria: (*i*) the restriction *P(r* = *0)* = *0*; (*ii*) *P(r)* should exhibit positive values; (*iii*) the *R_G_* from *GNOM* should agree with the ones derived from the Guinier analysis; and (*iv*) the curve should also be stable as *D_max_* is increased beyond the estimated maximal macromolecular length with *P*(*r*≥*D_max_*) = 0.

#### Scattering curves calculations and fitting

The scattering intensity *I(Q)* from particles in a solvent with scattering length *r_o_* and with an hydration shell of contrast *δρ* can be evaluated as:
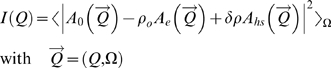



The particle has a scattering density *ρ_a_* and is surrounded by a solvent with an average scattering density of *ρ_o_*; the hydration shell is depicted by a border layer of effective thickness *D* and density *ρ_b_* different from *ρ_o_*. Here 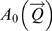, 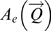 and 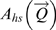 are the amplitudes from the particle *in vacuo*, the excluded volume and the hydration shell, respectively. Ω is the solid angle in reciprocal space. *I(Q)* is an average over all orientations of the particles in solution. The particle shape is described as an angular envelope function. The scattering from the hydration shell is simulated by surrounding the envelope function with a layer of thickness Δ = 3Å and density *ρ_b_*. Experimental curves *I_exp_(Q)* are fitted against calculated curves *I_calc_(Q)* by adjusting two parameters, the total excluded volume *V* and the contrast between of the border layer *δρ* = *ρ_b_*−*ρ_o_* to minimize the discrepancy *χ* defined as:
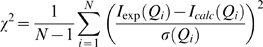

*N* is the total number of experimental points and *σ*(*Q_i_*), their associated standard deviations. In practice, theoretical scattering curves were calculated using *CRYSOL* [24] programs and automatically fitted against experimental curves. The resulting fitting parameters adjusted in *CRYSOL* corresponded to a reasonable description of the hydration properties of the particles in solution.

#### Ab initio three-dimensional reconstruction of molecular shapes

Low resolution shape retrieving from one-dimensional small angle X-ray scattering data in solution was performed using the program *DAMMIN* [26]. A Dummy Atom Model (DAM) is randomly generated and composed of an array of given contrast spheres densely packed on a hexagonal face-centered cubic lattice of given lattice constant. The scattering intensity from any given DAM is calculated by global summation over all dummy atoms using spherical harmonics expansion to represent partial amplitudes. *Ab initio* reconstruction consists in finding a DAM configuration corresponding to a minimal value of a goal energy function and minimizing the discrepancy between experimental and DAM-based scattering curves. Compactness, connectivity and looseness of the DAM are described by functions that are taken into account during the global minimization procedure that uses simulated annealing. The algorithm proceeds iteratively through a single dummy atom move. No shape constraints were introduced during the calculations. Ten independent reconstructions trials were performed for each receptor.

## Supporting Information

Figure S1 Conformational changes in the active site of FtsY. The apo and GDP•magnesium structures of Pfu-FtsY are superposed together with the GMPPCP-bound Taq-FtsY (as seen in the FtsY•FfhNG complex) and GDP-bound truncated Taq-FtsY. The GDP•magnesium (Pfu), anion (Pfu), GDP (Taq) and GMPPCP•magnesium (Taq) are shown in yellow, blue, orange and red respectively. Residues are numbered for Pfu and Taq (in italic). In the GDP-bound Taq-FtsY structure, Arg142 is on a disordered loop and was not seen. The conserved SRP-GTPase motifs I, II, IV and V are indicated.
(2.09 MB TIF)

Figure S2 Alignment of archeal FtsY sequences. The sequence of Pfu-FtsY is aligned against 12 FtsY sequences from archeons representative of all families constituting the archeal kingdom. The secondary structure elements of Pfu-FtsY are indicated. The sequences correspond to Pyrococcus furiosus, Sulfolobus solfataricus, Methanococcus jannaschii, Archaeglobus fulgidus, Thermococcus zilligii, Halobacterium salinarum, Pyrobaculum aerophilum, Methanothermobacter thermoautotrophicus, Methanoculleus, marisnigri, Aeropyrum pernix, Thermoplasma acidophilum and Methanosaeta thermophila. The alignment is restricted to the N domain. All sequences are truncated at the strictly conserved glycine residue (Gly130 in Pfu) delineating the start of motif I (the P-loop) in all SRP/SR GTPases and indicated by a red asterisk. Note the long insertion present in the N domain from Methanococcus. Note the extreme difference in size observed between the N domains from Methanococcus (200 residues) or Thermoplasma (88 residues). Sequence and domain-size variability mainly arises in the insertion located between region the αN1 and αN2 helices (blue line). The N-terminal end of the αN1 helix is characterized by a conserved phenylalanine residue and its high content in basic residues (red line).
(0.72 MB EPS)

Movie S1 A model for the association between the SRP and its SR in the targeting complex. The movie shows the model as presented in Figure 7. The N-terminal helices αN1 of both FtsY and SRP54 are highlighted (yellow) to emphasize their positions relative to the α7 helices (magenta) at the C-terminus of each G domain.
(4.77 MB MOV)
