# Supplementary figures and images for: Structures of the Signal Recognition Particle Receptor from the Archaeon Pyrococcus furiosus: Implications for the Targeting Step at the Membrane
Source: PLoS One. 2008 Nov 3;3(11):e3619. doi: 10.1371/journal.pone.0003619 (PMC2572998; doi:10.1371/journal.pone.0003619)

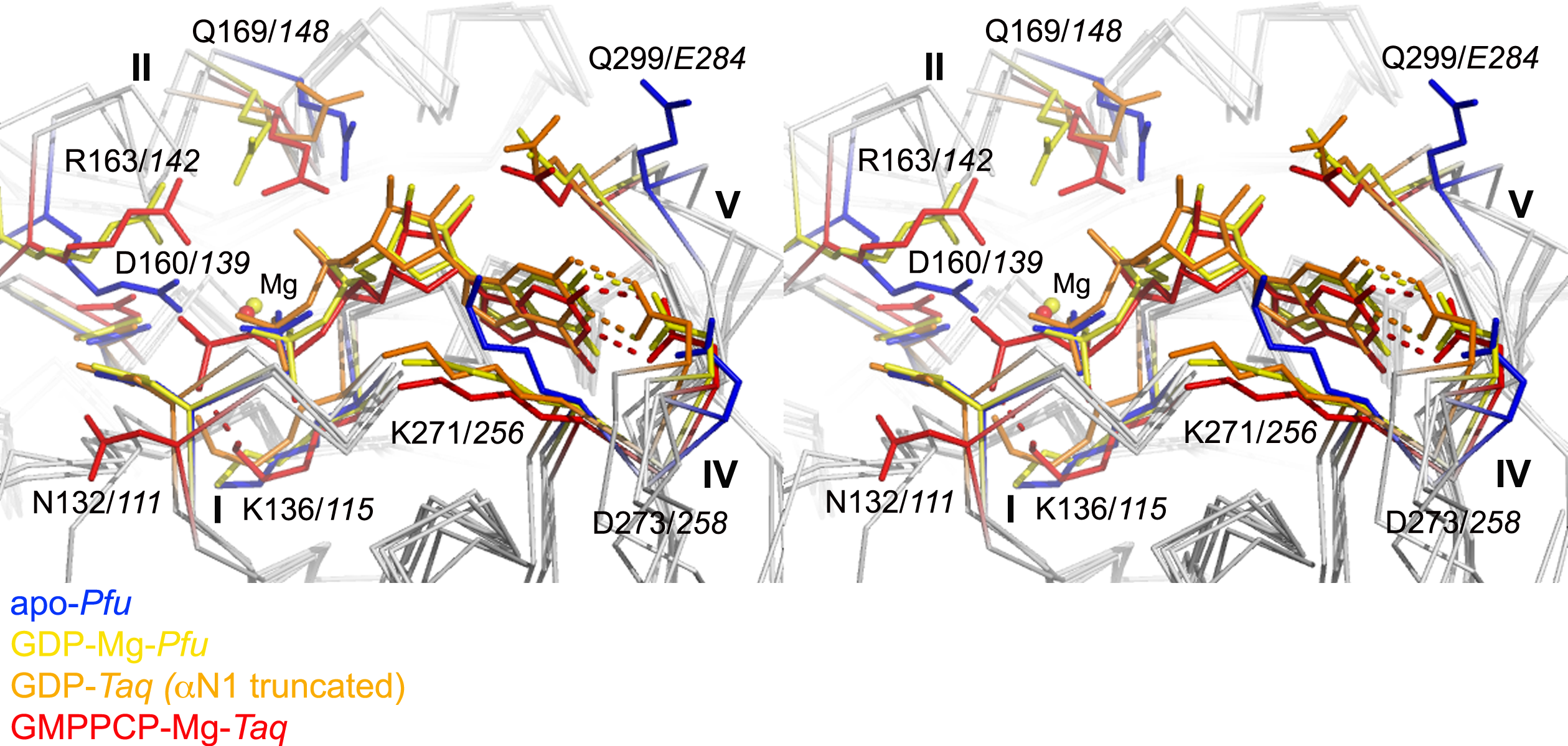

Supplement: Figure S1 — Conformational changes in the active site of FtsY. The apo and GDP•magnesium structures of Pfu-FtsY are superposed together with the GMPPCP-bound Taq-FtsY (as seen in the FtsY•FfhNG complex) and GDP-bound truncated Taq-FtsY. The GDP•magnesium (Pfu), anion (Pfu), GDP (Taq) and GMPPCP•magnesium (Taq) are shown in yellow, blue, orange and red respectively. Residues are numbered for Pfu and Taq (in italic). In the GDP-bound Taq-FtsY structure, Arg142 is on a disordered loop and was not seen. The conserved SRP-GTPase motifs I, II, IV and V are indicated. (2.09 MB TIF) [file pone.0003619.s001.tif]
